# Corrigendum: Efficacy and safety of darbepoetin alfa injection replacing epoetin alfa injection for the treatment of renal anemia in Chinese hemodialysis patients: A randomized, open‐label, parallel‐group, noninferiority phase III trial

**DOI:** 10.1002/cdt3.94

**Published:** 2023-09-19

In the article titled, “Efficacy and safety of darbepoetin alfa injection replacing epoetin alfa injection for the treatment of renal anemia in Chinese hemodialysis patients: A randomized, open‐label, parallel‐group, noninferiority phase III trial” published in pages 134‐144, issue 2, vol. 8 of Chronic Diseases and Translational Medicine,^1^ the information of trial registration at the end of Abstract is missing. The registration information should be: This study has been registered on www.chinadrugtrials.org.cn, registered number CTR20130079.
